# HLA-Haploidentical Family Donors: The New Promise for Childhood Acute Lymphoblastic Leukaemia?

**DOI:** 10.3389/fped.2021.758680

**Published:** 2022-01-21

**Authors:** Syaza Ab Rahman, Toni Matic, Maya Yordanova, Hany Ariffin

**Affiliations:** ^1^Paediatric Haematology-Oncology and Bone Marrow Transplantation Unit, University of Malaya Medical Centre, Kuala Lumpur, Malaysia; ^2^Department of Paediatrics, University Hospital Centre, Zagreb, Croatia; ^3^Children's Oncohematology Unit, Queen Johanna University Hospital, Sofia, Bulgaria

**Keywords:** haploidentical, haematopoietic stem cell transplantation, paediatric, acute lymphoblastic leukaemia (ALL), human leukocyte antigen

## Abstract

Allogeneic haematopoietic stem cell transplantation (HSCT) is indicated in children with high-risk, relapsed or refractory acute lymphoblastic leukaemia (ALL). HLA-matched grafts from cord blood and stem cell repositories have allowed patients without suitable sibling donors to undergo HSCT. However, challenges in procuring matched unrelated donor (MUD) grafts due to high cost, ethnic disparity and time constraints have led to the exponential rise in the use of stem cells from human leukocyte antigen (HLA)-haploidentical family donors. Whilst HLA-haploidentical HSCT (hHSCT) performed in adult patients with acute leukaemia has produced outcomes similar to MUD transplants, experience in children is limited. Over the last 5 years, more data have emerged on hHSCT in the childhood ALL setting, allowing comparisons with matched donor transplants. The feasibility of hHSCT using adult family donors in childhood ALL may also address the ethical issues related to selection of minor siblings in matched sibling donor transplants. Here, we review hHSCT in paediatric recipients with ALL and highlight the emergence of hHSCT as a promising therapeutic option for patients lacking a suitable matched donor. Recent issues related to conditioning regimens, donor selection and graft-vs.-host disease prophylaxis are discussed. We also identify areas for future research to address transplant-related complications and improve post-transplant disease-free survival.

## Background

Allogeneic haematopoietic stem cell transplantation (HSCT) is used to consolidate remission in patients with genetic subtypes of childhood acute lymphoblastic leukaemia (ALL) at high risk of relapse as well as those with relapsed or refractory disease. For the latter two groups, immunotherapy such as anti-CD19 antibodies as well as chimeric antigen receptor (CAR) T cells have been utilised. However, these new immunotherapy modalities are relatively expensive and not universally available. Notably, immunotherapy has not completely removed the need for HSCT in patients with relapsed or refractory ALL.

Currently, human leukocyte antigen (HLA)-matched sibling donors (MSDs) are the preferred choice for children with ALL who need to undergo HSCT for disease control (1). However, sibling pairs have only a 25% chance of inheriting the same HLA haplotype; thus, volunteer donor stem cell and umbilical cord blood registries have been established to provide an alternative source of HLA-matched donor grafts.

Banked cryopreserved umbilical cord blood units allow greater mismatching degree and are easily available with faster procurement. Studies have demonstrated the benefits of umbilical cord blood transplantation in paediatric haematologic malignancies where cord blood cell dose and HLA-grade matching are crucial factors for transplant outcome (2–4). However, umbilical cord blood transplantation still carries high risks of graft failure, delayed engraftment and slower immune reconstitution. Additionally, with single umbilical cord blood units, there is no source for subsequent stem cell boost or other cell-based therapies (4).

Despite the availability of international stem cell repositories, challenges in procuring matched unrelated donor (MUD) grafts due to high costs, ethnic disparity and time constraints have led to the exponential rise in the use of stem cells from HLA-haploidentical family donors, reflected in registry data of the last decade (5). HLA-haploidentical HSCT (hHSCT) allows immediate and almost universal family donor availability (HLA-matched at 8 out of 10 loci or less) at lower cost and easier accessibility than MUD and so has expanded curative options for many ALL patients with urgent transplant indications.

Currently, hHSCT can be performed using either manipulated or unmanipulated grafts with various strategies to eliminate prohibitive graft-vs.-host disease (GvHD). For manipulated donor marrow or peripheral blood grafts, *ex-vivo* T-cell depletion is performed using sophisticated cell sorting machines which remove immune cell subsets that cause GvHD (TCRαβ, CD45RA^+^, CD19^+^ depletion). Conversely, subsets that provide graft-vs.-leukaemia (GVL) effect namely TCRγδ T cells as well as NK cells, monocytes and dendritic cells which promote prompt immune reconstitution are retained.

A technically simpler platform comprises an unmanipulated graft with *in vivo* depletion of alloreactive T cells and high-dose post-transplant cyclophosphamide (PTCy). The hHSCT-PTCy technique—pioneered by researchers from Johns Hopkins University (6)—is widely applied clinically and has substantially extended the use of hHSCT in patients with acute leukaemia. Another method is the Beijing “GIAC” protocol, developed by Huang et al. This comprises granulocyte colony-stimulating factor (G-CSF)-primed donor peripheral blood and marrow stem cells and intensive immunosuppression using mycophenolate mofetil (MMF), cyclosporine A (CsA), methotrexate and anti-thymocyte globulin (ATG) (7).

Here, we review the hHSCT experience for children with ALL and discuss the development of hHSCT as a promising therapeutic option for those lacking an HLA-matched donor.

## Immunobiology Considerations in HSCT

HLA diversity is the cornerstone of “self” vs. “foreign” recognition in the immune system. The biological role of HLA class I and class II molecules is to present processed peptide antigens to immune cells for non-self recognition and killing. HLA mismatch between a recipient and a stem cell donor represents a bi-directional risk factor for both GvHD and graft rejection (host-versus-graft). GvHD is caused by immunocompetent donor T cells contained in the stem cell graft. In hHSCT, several methods have been developed to deplete alloreactive donor T cells with the goal of averting or minimising GvHD. However, although efficient T-cell depletion of donor marrow leads to a lower incidence of acute GvHD, a higher incidence of graft failure, leukaemia relapse and delayed immune reconstitution may result (8). Graft rejection in this instance is mediated by recipient cytotoxic T lymphocyte precursors that survive the conditioning regimen, along with anti-donor HLA antibodies (9, 10). Although markedly reduced by pre-transplantation conditioning chemotherapy or radiation, residual recipient immune cells are often adequate to mount a response against a graft that is “unprotected” by donor immune cells. Thus, a successful hHSCT outcome requires a nuanced immunological balance between the haploidentical graft and recipient.

T cells play a central role in the pathophysiology of both GvHD and the GVL effect. A key event in the development of acute GvHD is the interaction of T cells expressing a suitable T-cell receptor with antigen-presenting cells that express host major histocompatibility complex or minor histocompatibility antigen peptides. Activated CD8^+^ T-cytotoxic and CD4^+^ T-helper (Th)1, Th2, and Th17 cells can directly cause GvHD *via* release of cytolytic cytokines such as perforin or tumour necrosis factor alpha (11). Additionally, co-stimulatory pathways such as CD40 ligand (12) and programmed death 1 and programmed death ligand 1 (PD-L1) (13)—key cytokines that influence T-cell differentiation as well as metabolic pathways that provide energy for T-cell proliferation (14)—contribute to overlapping mechanisms that promote GvHD. In gut GVHD, intestinal tissue damage from conditioning therapy results in the recruitment of innate immune cells to the injured tissue and release of damage-associated molecular pattern (DAMP) molecules. Infiltration of neutrophils and monocytes into the gastrointestinal tract causes activation and production of reactive oxygen species that contribute directly to tissue damage. DAMP molecules enhance GVHD through cleavage of precursor intracellular cytokine pro-interleukin-1β into its bioactive form by caspase-1 or caspase-11, and through the transcription of genes that encode cytokines and chemokines that promote GVHD. Inflammatory responses may also be induced by infectious pathogens that trigger the release of pathogen-associated molecular pattern (PAMPs) molecules. These molecules activate innate immune cells that migrate from damaged intestinal epithelium to mesenteric lymph nodes for antigen presentation and donor T cell activation (14).

Recently, two groups (15, 16) have proposed a novel mechanism of GvHD pathophysiology. Using single-cell analysis, Jardine et al. demonstrated that acute GvHD can result from peripheral host T cells resident in the skin and gut being stimulated against donor antigen-presenting cells in the form of monocyte-derived macrophages. These donor-derived macrophages have enhanced antigen-presenting functions that could enable the activation of residual host T cells, resulting in host-vs.-graft responses that may be indistinguishable from GvHD clinically (16). Divito et al. reported similar findings of host peripheral T cells in skin GvHD specimens (15). They developed a humanised mouse model of skin GvHD where skin-resident host T cells were activated by donor monocytes (15).

CD4^+^/CD25^+^/Foxp3^+^ regulatory T cells (Tregs) play a protective role by downregulating the immune response when it is no longer needed, thus maintaining immune homeostasis. Tregs suppress the immune response in several ways, including: (1) producing anti-inflammatory cytokines such as transforming growth factor (TGF)-b and interleukin (IL)-10; (2) suppressing activation and proliferation of both T-helper and T-cytotoxic cells; and (3) suppressing B cells and dendritic cells. Memory CD45RO^+^ Tregs do not express the bone marrow homing receptor CXCR4; thus, few donor Tregs migrate to the host marrow (17, 18). The lack of donor Tregs in the marrow allows for unopposed conventional T-cell alloreactivity and is the basis for the GVL effect.

NK cells are regulated by a number of receptors that finely tune potent effector functions including cytolytic activity against different target cells and release of cytokines that play a major role in inflammation and immunoregulation. NK-cell education or licencing facilitates a balance between self-tolerance under physiologic conditions and maintenance of the ability to mediate an immune response against microbial pathogens and leukaemia cells (19). The role of natural killer (NK) cells in the pathogenesis of GVHD is still controversial. The conventional view is that, in contrast to T cells, alloreactive NK cells protect against GvHD. Normal recipient tissues that are common targets of T-cell-mediated GvHD, such as skin and gut mucosa, are spared due to lack of ligands for activating NK-cell receptors. Donor NK cells can also eliminate recipient-type antigen-presenting cells, a process that is based on mismatches of killer-cell immunoglobulin-like receptors (KIR), thus preventing presentation of host antigens to graft T cells. However, several recent studies have revealed that whilst NK cells naturally suppress GVHD, highly pre-activated NK cells can induce donor T-cell alloreactivity through the production of proinflammatory cytokines such as TNF-α and IFN-γ (20).

NK-cell alloreactivity is especially useful in the setting of HLA-mismatched transplants, where NK cells exert anti-leukaemic activity without concomitant GvHD (21). In HLA-mismatched HSCT for acute myeloid leukaemia (AML), NK-cell alloreactivity has been shown to decrease the risk of relapse while enhancing engraftment and reducing GvHD by eliminating host dendritic cells (22). More recently, improved understanding of NK cell biology has led to the use of KIR-ligand mismatched donors to enhance the GVL effect in patients undergoing HSCT for haematologic malignancies (23, 24).

The goal of hHSCT is to optimally manipulate immune cells of both the host and donor to achieve stable engraftment, immune reconstitution with adequate GVL effect and protection against infections, while simultaneously achieving immune tolerance which affords acceptable GvHD.

## hHSCT in the Treatment of Haematological Malignancies

hHSCT has been utilised in the management of patients with haematological malignancies for over 30 years. In the 1990's, a group in Perugia successfully demonstrated a method to overcome the immunological barrier in hHSCT through the infusion of “megadose” T-cell-depleted progenitor cells after high-intensity conditioning in adults with acute leukaemia (25). The method was associated with high engraftment rates and minimal GvHD but a high incidence of non-relapse mortality (NRM) and relapse rates (26). Over time, optimization of the conditioning regimen and evolving graft processing techniques for modulation of T-cell alloreactivity have alleviated the main challenges for transplantation across the HLA barrier, i.e., graft rejection, GvHD and unacceptably high treatment-related toxicity (TRM).

### T-Cell-Depleted hHSCT

Introduction of refined, partial T-cell-depletion methods (αβ-depleted hHSCT) has considerably improved post-transplant immune reconstitution as well as anti-infective and anti-leukaemia (GVL) activity, resulting in outcomes comparable to MSD and MUD transplants (27–30).

In a single-centre cohort of 80 children with ALL in remission, Locatelli et al. reported a disease-free survival (DFS) of 71% using TCRαβ T-cell- and CD19-depleted hHSCT following myeloablative conditioning with ATG, comparable to DFS with transplants using MSD or MUD grafts (30). Another Italian multicentre study that involved 98 children with leukaemia who underwent TCRαβ T-cell- and CD19-depleted hHSCT following myeloablation presented a 5-year probability of leukaemia-free survival (LFS) of 62%, with chronic GvHD-free/relapse-free survival (GRFS) outcomes comparable to those of MUD transplants and superior to mismatched unrelated donor (MMUD) transplants (27). Lang et al. reported encouraging results for children with leukaemia in first complete remission (CR1) to CR3 using TCRαβ T-cell- and CD19-depleted hHSCT following myeloablation (1-year EFS of 100%), although no patients with active disease survived (29). This group also showed the successful use of TCRαβ T-cell- and CD19-depleted hHSCT following reduced-intensity conditioning (RIC) in a study of 30 patients, including 10 patients with ALL (31). Good outcomes using T-cell-depleted hHSCT were also reported by Shelikhova et al. in paediatric ALL patients; the probability of 2-year EFS was 49.6% and 2-year OS was 50%) (32). A Turkish study in paediatric acute leukaemia patients showed that the survival of patients with high-risk acute leukaemia after TCRαβ T-cell- and CD19-depleted hHSCT with use of ATG and mesenchymal stem cells was comparable to MUD transplantation (28). Jacoby et al. reported an EFS of 61% in children with leukaemia using total body irradiation (TBI)-based conditioning and αβ-T-cell-depleted hHSCT (33). Recently, the ALL SCT Berlin-Frankfurt-Münster (BFM) Study Group conducted a study with 569 children with very-high-risk ALL who received HSCT. Among them, 106 patients had a graft from a mismatched donor and 62 of them received an *ex vivo* T-cell-depleted peripheral blood stem cell graft either by positive CD34^+^ selection or by negative CD3^+^/CD19^+^ depletion. The 4-year EFS was statistically better for patients transplanted from matched compared with mismatched donors and this was attributed to a lower NRM (1).

### T-Cell Replete hHSCT With PTCy

The PTCy method of hHSCT has been used with both RIC and myeloablative conditioning regimens and also using either blood or marrow stem cells. In the original PTCy-based protocol published by the John Hopkins University group, the use of RIC with PTCy after bone marrow transplantation was associated with acceptable incidences of graft failure and GvHD but a high risk of relapse (6). There is limited data to estimate the efficiency and safety of hHSCT using unmanipulated grafts in the paediatric setting and results from larger studies of adult patients with ALL may not be accurate to extrapolate to children. In several small series of T-cell-replete hHSCT in high-risk paediatric ALL patients, acceptable rates of GvHD and NRM with effective and rapid immune reconstitution have been reported (34, 35).

The first study in a paediatric-only cohort was from Japan and comprised 15 children with advanced leukaemia (36). Both bone marrow and peripheral blood grafts were used. CR was achieved in 46% of the patients but long-term outcome was poor. A high incidence of grade III–IV GvHD (25%) was reported and this was attributed to the use of RIC and single-day cyclophosphamide (day +3) as opposed to two doses. Klein et al. studied the use of RIC in children and young adults with haematological malignancies, including ALL, and found a low NRM rate but a high cumulative incidence of relapse (52% at 2 years) (37). Majority of patients received bone marrow graft and two patients received peripheral blood graft. Another recent study by Trujillo et al. reported on 42 children with high-risk malignancies (22 with ALL) who underwent hHSCT-PTCy with RIC (fludarabine plus busulfan or melphalan, and low-dose TBI) and peripheral blood as the stem cell source (38). The group demonstrated outcomes comparable to studies utilising myeloablative regimens, with 1-year TRM of 14%, a relapse rate of 26%, 3-year OS of 56%, and 3-year EFS of 46%. However, a high incidence of moderate-to-severe GvHD was seen in younger children, with 40% of those <10 years of age experiencing grade III–IV GvHD. In a retrospective study comparing hHSCT-PTCy to HSCT using an MUD or MMUD after RIC in paediatric patients with acute leukaemia, a group in Italy reported similar outcomes with regards to 5-year OS, NRM and relapse incidence between the three groups (39).

A myeloablative preparative regimen followed by hHSCT-PTCy using peripheral blood stem cells was used in 20 children with advanced leukaemias. The 2-year OS as reported by Jaiswal et al. was 64.3% (40). NRM at 1 year was 20% and this was associated with grade III–IV GvHD (39). Similarly to the study by Trujillo et al., it was noted that high-grade GvHD occurred only in children <10 years and there was a higher incidence of early alloreactivity in the form of haemophagocytic syndrome in this age group, findings not previously noted in the adult population. Other studies which have used myeloablation followed by hHSCT-PTCy involving children with ALL were by Uygun et al. (*n* = 60, Turkey), Yesilipek et al. (*n* = 15, Turkey) and Symons et al. (*n* = 96, USA). The 1-year OS for these children was 64, 75, and 73%, respectively, whilst EFS was 59, 68, and 57%, respectively (41–43). Katsanis et al. conducted a study utilising hHSCT- PTCy – this time in 13 ALL patients who received myeloablation and were negative for minimal residual disease (MRD) prior to hHSCT. With a median follow-up of 25 months, OS was 84.0% and the GRFS rate was 50.1% (44).

An Italian study in 33 children with haematological malignancies (15 with ALL) using RIC or myeloablative conditioning mostly with bone marrow stem cell graft reported 1-year OS of 72%, CIR of 24%, and TRM of 9% (34). In a similarly designed study by Sharma et al. (17 children with acute leukaemia, median follow-up of 393 days, use of peripheral blood stem cells), OS and EFS were 70.5 and 64.7%, respectively. Of note, three of four children who received RIC relapsed (45).

Recently and on behalf of the European Society of Blood and Marrow Transplantation (EBMT), Ruggeri et al. reported on 180 children with ALL (69% in CR1 or CR2) who received a preparative regimen of either myeloablative conditioning or RIC (46). The results were promising, with a cumulative incidence of relapse (CIR) of 25.1 and 37% for those in CR1 and CR2, respectively, and 2-year NRM of 19.6% for the whole cohort. Cumulative incidence of grade III–IV aGvHD was 12.4%, with a worse outcome in those who received peripheral blood stem cells compared with bone marrow grafts (18.9 vs. 6.2%, respectively; *p* = 0.04). Disease status was an independent predictor of reduced survival, with 2-year LFS of 65, 44, and 18.8% in those transplanted in CR1, CR2, and CR3, respectively, and 1-year LFS of 3% for those transplanted in active disease.

More recently, several groups have adopted modifications to the hHSCT-PTCy approach. Adaptations include earlier initiation of a calcineurin inhibitor (CsA or tacrolimus) on day 0 and MMF on day +1 followed by PTCy on days +3 and +5 (47, 48). Early administration of a calcineurin inhibitor is thought to spare some donor lymphocytes from the tolerizing effects of cyclophosphamide, thus preserving a GVL effect and reducing the incidence of relapse (49). Previous studies have found this modified approach to be associated with low rates of chronic GvHD and a CIR of about 25% in adult patients with haematologic malignancies (47, 48). The Acute Leukaemia Working Party-EBMT group recently published a retrospective comparative study on the timing of PTCy and immunosuppressive therapy in 509 patients with acute leukaemia. When compared with patients who received PTCy on days +3 and +4 along with CsA/tacrolimus + MMF on day +5, the group who received PTCy on days +3 and +5 with early CsA + MMF initiation on days 0 and +1, respectively, demonstrated significantly better LFS (HR 0.62; *p* = 0.02) and GRFS (hazard ratio [HR] 0.58; *p* = 0.02) primarily due to a lower incidence of relapse (50).

Overall, these recent studies validate the feasibility of the hHSCT-PTCy platform for children with high-risk ALL. The optimal timing for cyclophosphamide administration and the combination of immunosuppressive agents in hHSCT is still unknown, although several studies have shown encouraging outcomes for the modified PTCy approach (42, 47, 48, 50).

### ATG-Based T-Cell Replete hHSCT

Application of the Beijing “GIAC” hHSCT protocol in 42 children with haematological malignancies was first reported by Liu et al. (51). Outcomes were acceptable, with 3-year LFS of 57.3%, but there were high rates of acute grade II–IV GvHD (57.0%) and chronic GvHD (56.7%) (51). Five years later, the same group reported on the efficacy and safety of this transplantation method for children with ALL and acute myeloid leukaemia (AML) in CR1 or CR2 (52). The 5-year LFS for patients with ALL in CR1, CR2 and beyond CR2 or non-remission were 68.9, 56.6, and 22.2%, respectively. In this study, 19% of cases in CR1 relapsed, whilst NRM was 15% in CR1/CR2. A large study of 1,210 transplants by Wang et al. included children with ALL (450 patients <20 years; 38% patients with ALL) and reported DFS of 67% and NRM of 17% (53). A similar study by Mo et al. using the Beijing protocol in 65 children with high-risk ALL reported a 2-year probability of OS and DFS of 82 and 71%, respectively (54). Good results with an estimated 3-year OS of 69.5% and DFS of 63.5% have been noted in children with high-risk Philadelphia chromosome positive (Ph^+^) ALL managed with this hHSCT protocol (55, 56).

Several groups have described other methods of hHSCT without *ex-vivo* T-cell depletion and using ATG-based GvHD prophylaxis. A study by Ji et al. described the use of ATG-based GvHD prophylaxis with GCSF-primed bone marrow alone, and intensive post-transplant immunosuppression consisting of MMF, CsA, methotrexate, and the addition of anti-CD25 antibody, basiliximab, in 38 patients (both children and adults) with haematological malignancies. Basiliximab is a chimeric monoclonal antibody directed against CD25 present on activated lymphocytes and inhibits IL-2 mediated T cell activation and proliferation, thus reducing the risk of GVHD. The reported 2-year DFS was 53%, with low rates of acute grade II–IV GvHD (11%) and chronic GvHD (15%) (57). A similar study conducted by an Italian group reported very low rates of advanced and chronic GvHD—-at 5 and 6%, respectively—-but with a non-negligible TRM rate of 30%. The 3-year OS probability was 45% (54% in the standard-risk group, 33% in the high-risk group) and 3-year DFS was 38% (44% in the standard-risk group, 30% in the high-risk group) (58).

An innovative approach of combining the Beijing protocol with low-dose PTCy (14.5 mg/kg) in hHSCT has been reported in 114 patients with haematological malignancies who also had a high risk of post-transplant GvHD (mother or collateral donor). The study reported significantly improved incidences of grade III–IV GvHD (5 vs. 8%, *p* = 0.003), and improved NRM (6 vs. 15%, *p* = 0.045) compared with the original Beijing protocol (59), thus suggesting a synergistic combination of the two modalities.

Different registries have published reports on comparisons of outcomes between PTCy and the Beijing protocol in adult patients with leukaemia. In 2017, the EBMT consortium reported comparable outcomes in relapse rates and OS between PTCy and the Beijing protocol although NRM was lower in the PTCy arm (60). In contrast, the Chinese Bone Marrow Transplantation Registry Group (CBMTRG), reported significantly higher NRM and inferior PFS and OS in hHSCT-PTCy for haematological malignancies compared with G-CSF/ATG (61).

Direct comparison of outcomes using the different hHSCT approaches is difficult as studies in children have mostly involved small numbers of patients and included patients with other diagnoses. A head-to-head study comparing hHSCT using T-cell-depleted or T-cell-replete grafts in children with high-risk haematological malignancies was performed in Uruguay, involving 40 patients (15 with ALL) (62). T-cell-depleted transplants were performed using RIC, while most of those in the T-cell-replete PTCy arm received myeloablation. The results were comparable (actuarial OS rates at 2 years 47 vs. 48%, and 1-year TRM 24 vs. 26%, respectively, for the T-cell-depleted vs. T-cell-replete PTCy grafts) except for the incidence of chronic GvHD which was significantly higher in the PTCy group (9 vs. 53%, respectively, *p* = 0.029). In a larger study involving 192 children with high-risk leukaemia, the Spanish Working Group (GETMON/GETH) compared outcomes between hHSCT using PTCy and *ex-vivo* T-cell depletion. Similar OS, DFS and relapse incidence was observed between the two platforms, suggesting efficacy of both methods in childhood leukaemia (63).

A very comprehensive review and summary of the advantages and disadvantages of the three different approaches used in paediatric hHSCT has been done by Shah (64) and are summarized in Table 1. The Beijing GIAC method has the lowest risk of graft failure but has two disadvantages: namely a higher risk of GvHD and the need for the donor to undergo two stem collection procedures. Notably, GIAC hHSCT in children with haematological malignancies in CR1 has resulted in superior outcomes compared with transplants utilising umbilical cord blood from MUDs (65, 66). The John Hopkins' PTCy approach is easily applicable and has the lowest delivery cost yet carries a risk of graft failure risk of up to 15% as well as risks of sinusoidal obstruction syndrome (up to 20%) and haemorrhagic cystitis (up to 35%) (35, 37, 64). T-cell-depleted HSCT is associated with a very low incidence of GvHD but has been reported to have a higher risk of viral infections. Moreover, *ex vivo* T-cell depletion is costly and requires sophisticated laboratory infrastructure.

**Table 1:** Comparative features of the various hHSCT approaches used in treatment of children with haematological malignancies.

	**TCRαβ-depleted**	**PTCy**	**GIAC**
Conditioning	MAC or RIC	MAC or RIC	MAC
Stem cell source	PB	BM or PB	BM and PB
GVHD risk	Low	Low with BM Moderate with PB	High
Graft failure risk	Low	Moderate	Low
Cost	High	Low	Low/Moderate
Applicability	Sophisticated infrastructure needed	Easy	Easy
Viral infection risk	High	Moderate	Moderate

*BM, bone marrow; GIAC, GCSF-Intensive immunosuppression-ATG-Combined stem cell source (Beijing protocol); GVHD, graft-vs.-host disease; MAC, myeloablative conditioning; PB, peripheral blood; PTCy, post-transplantation cyclophosphamide; RIC, reduced-intensity conditioning; TCRαβ, T-cell receptor αβ*.

The various studies discussed above have demonstrated that hHSCT is efficacious in children with ALL. Whilst some studies have demonstrated that matched donor HSCT has superior CIR and NRM in children with ALL, these differences are largely seen only in those in the very-high-risk category (1). Thus, hHSCT remains a feasible transplant option for children with ALL lacking a matched donor, although infections and GvHD remain significant challenges.

## Optimal Donor Choice in hHSCT

For the majority of children, two or more potential haploidentical donors are usually available. Studies using various hHSCT methods have reported dissimilar incidence of GvHD, TRM and relapse incidence using different preferred donors, thus raising the question of best donor choice for a specific hHSCT platform. Here, we review studies involving only children with ALL as well as studies in which adult ALL patients or children with various other haematologic malignancies were included to identify the best donor option for each hHSCT method.

A large study of 1,210 patients treated using the Beijing hHSCT platform in children and adults with haematological malignancies including ALL was conducted by Wang et al. (53). Younger donors and paternal donors were associated with better outcomes (lower NRM and better survival) when compared with older donors and maternal donors, respectively.

Transplants using sibling donors who did not share inherited maternal HLA antigens with the recipient (i.e., non-inherited maternal antigen [NIMA] mismatched) were associated with the lowest incidence of acute GvHD when compared with transplants using sibling donors who were non-inherited paternal antigen (NIPA) mismatched or parental donors. Thus, for hHSCT using the Beijing protocol, a NIMA-mismatched younger male sibling is the preferred donor followed by the father over the mother or a sister.

Optimal donor choice for children with ALL undergoing hHSCT-PTCy has largely been extrapolated from studies using adults, although some studies have included paediatric patients. These studies have alluded to an influence of donor age and gender on outcomes. Berger et al. reported that in 33 children and adolescents with various haematological malignancies who underwent hHSCT-PTCy, female patients and patients who had maternal or other female donors had a significantly lower risk of relapse than other patients (female vs. male patient: 7 vs. 35%; female donor vs. male donor: 10 vs. 40%; mother donor vs. other donor: 0 vs. 35%, respectively) (34). In contrast, Kasamon et al. found that hHSCT-PTCy in male recipients with a female donor was associated with an inferior EFS compared with male recipients with a male donor (HR 1.47; *p* = 0.04) (67). More recent paediatric studies have not confirmed selection criteria for the most ideal donor. However, donors of the same sex and with a similar ABO blood group and cytomegalovirus serostatus as the recipient are preferred, as are recipients with an absence of HLA antibodies to the donor. In an international study of 180 children with ALL who received hHSCT-PTCy, a multivariate analysis found that donor selection based on relationship to recipient did not affect NRM; instead, disease status at transplant, age >13 years and use of peripheral blood stem cell grafts were independent factors associated with decreased OS (46). Trujillo et al. reported on 26 children with ALL who received RIC followed by hHSCT-PTCy. The incidence of acute grade III–IV GvHD was 17%, OS was 56%, and EFS was 46%, with no association between these outcomes and donor–recipient kinship (38).

The impact of donor selection has been more thoroughly investigated in hHSCT using T-cell-depleted grafts. In a study of 36 paediatric patients (17 AML, 19 ALL) who received haploidentical T-cell depleted (CD34^+^ selected) grafts, the risk of relapse was best predicted by the presence of inhibitory KIR on the donor's NK cells and the absence of matching KIR ligand in the HLA repertoire of the recipient (68). In contrast to previously described ligand–ligand models, this was named a receptor–ligand model (or missing-self model); NK-cell alloreactivity based on this model more accurately predicted a lower risk of relapse. Additional factors that confer a reduced risk of relapse in children with ALL include the use of grafts from KIR haplotype B donors compared with KIR haplotype A, and the presence of centromeric but absence of telomeric group B KIR haplotypes (69, 70). Taken together, these studies suggested that KIR genotyping is an important consideration for donor selection in T-cell-depleted hHSCT. Donor age and sex have also been recognised to influence transplant outcomes in this setting. A study of 94 paediatric patients with high-risk leukaemia who received CD3^+^/CD19^+^ and TCRαb^+^/CD19^+^ T-cell-depleted haploidentical grafts by Gonzalez-Vicent et al. demonstrated faster recovery of immune cells as well as lower NRM when using donors <40 years old (NRM: donor >40 years, 43%; donor <40 years, 13%; *p* = 0.006) (71). With regards to donor sex, a retrospective analysis of 118 patients with acute leukaemia which also included children who received T-cell-depleted hHSCT after myeloablative conditioning by Stern et al. showed that donor sex in parental donor transplantation is an independent prognostic factor for survival (HR for father vs. mother 2.36; *p* = 0.003) (72). However, donor sex had no influence on survival if the donor was a sibling. These data suggested a mother should be preferred as the parental donor in T-cell-depleted hHSCT.

In 2019, the EBMT published consensus recommendations for donor selection in hHSCT based on a comprehensive review of literature combining adult and childhood subjects (73). A summary of the recommendations provided for the two broad hHSCT groups, namely T-cell-depleted and T-cell-replete hHSCT (including PTCy and Beijing platforms), is shown in Table 2.

**Table 2:** EBMT consensus recommendations for donor selection in hHSCT.

**T-cell-depleted hHSCT**	**T-cell-replete hHSCT**
1. For a recipient with donor-specific anti-HLA antibodies, a donor without the corresponding HLA antigen is preferred (MFI <1,000) 2. NK-cell alloreactive donor if available 3. Younger donor over older donor 4. A male donor for a male recipient 5. First-degree relative over second-degree HLA-half-matched donor 6. Between parent donors, mother is preferred over father 7. ABO-matched donor 8. CMV-seropositive donor for CMV-seropositive recipient	1. For a recipient with donor-specific anti-HLA antibodies, a donor without the corresponding HLA antigen is preferred (MFI <1,000) 2. Younger donor over older donor 3. A male donor for a male recipient 4. Sibling or offspring donor over parent donor 5. Between parent donors, father is preferred over mother donor 6. An ABO-matched donor is preferred to a minor ABO-mismatched donor, and a minor ABO-mismatched donor is preferred to major ABO-mismatched donor. 7. First-degree relative over second-degree HLA-half-matched donor (Beijing protocol) 8. Donor with KIR ligand match (Beijing protocol) 9. Donor with NIMA mismatch over NIPA mismatch (Beijing protocol)

*Table modified from Ciurea et al. (73). MFI, mean fluorescence intensity; NK, natural killer; NIMA, non-inherited maternal antigens; NIPA, non-inherited paternal antigens*.

One ethical issue which remains to be resolved is the use of sibling donors who are minors (aged <18 years). This situation provides a potential conflict of interest for parents. Regulations are different between countries; in some countries, relevant laws do not exist. International standards published by the Foundation for the Accreditation of Cellular Therapy at the University of Nebraska Medical Centre (FACT) and Joint Accreditation Committee of the International Society for Cell and Gene Therapy and EBMT (JACIE) suggest using donor advocates who are not the transplant recipient's treating physician to represent the minor donors (74). The advocate would help the donor to understand the risks and benefits of stem cell donation, try to resolve potential medical and psychological problems and obtain consent to donate without any pressure. A medical ethicist may also be involved to provide an unbiased assessment.

Related to this, grafts from HLA-haploidentical second-degree related donors (namely aunts, uncles and cousins) have also been successfully used for hHSCT involving both PTCy and Beijing approaches (75, 76). This is a feasible option if no suitable first-degree relative is available (77) and may help to address ethical conflicts related to using minor siblings as donors.

## Optimal Choice of Conditioning Regimen in hHSCT

Different types of preparative regimens for the various hHSCT platforms have been proposed. Myeloablative conditioning has been more frequently utilised with the Beijing protocol and TCRαβ-CD19-depleted hHSCT approaches vs. RIC for patients with haematological malignancies. In the original hHSCT-PTCy method for adults with leukaemia, RIC was used but later studies employed myeloablative conditioning with better EFS and no significant increase in NRM or GvHD (64).

Several studies in children with ALL comparing TBI-based and chemotherapy-based myeloablation in the haploidentical setting have been published, and the results are mostly in favour of TBI. In the aforementioned study of 80 children with acute leukaemia who received myeloablation and TCRαβ T-cell- and CD19-depleted hHSCT, Locatelli et al. reported that the use of TBI was associated with reduced incidence of relapse and better GRFS compared with the use of chemotherapy-based conditioning (30). In a study of 18 patients with high-risk paediatric haematological malignancies who underwent TCRαβ-depleted hHSCT, patients conditioned with TBI had superior OS (66 vs. 37%, respectively; *p* = 0.05) and EFS (61 vs. 33%, respectively; *p* = 0.04) compared with patients conditioned with chemotherapy only (33).

In another study, involving 42 children with ALL who received TCRαβ-depleted hHSCT, those who received TBI-based conditioning had a trend towards better EFS compared with those given treosulfan-based myeloablation (62.0 vs. 46.5%, respectively), although this result did not reach statistical significance (32). In contrast to the above studies, Bertaina et al. reported on a study including 98 Italian children who received TCRαβ T-cell- and CD19-depleted hHSCT; the type of myeloablative regimen employed (TBI based or chemotherapy based) did not influence LFS (27). To address the question of the best choice of conditioning for children with high-risk ALL, the For Omitting Radiation Under Majority age (FORUM) trial was launched in mid-2013. FORUM was a randomised, controlled, open-label multicentre trial involving 417 children with high-risk ALL who received myeloablative allogeneic HSCT. Among patients aged >4 years who received HLA-matched grafts, superior OS (91 vs. 75%, respectively; *p* < 0.0001) and lower relapse risk (12 vs. 33%, respectively; *p* < 0.0001) were observed in those conditioned with myeloablative TBI plus etoposide compared with those receiving myeloablative chemoconditioning (thiotepa and fludarabine with either busulfan or treosulfan) (78). Patients who received HLA-mismatched donor grafts, including from haploidentical donors or MUDs, were also observed. Preliminary results for this latter group of patients did not show any significant difference between TBI-based conditioning vs. chemo-conditioning with regard to OS, EFS, CIR or TRM, but final results are yet to be determined. In the series by Ruggeri et al. involving 139 children with ALL who received myeloablation followed by hHSCT-PTCy, relapse incidence at 2 years was higher in those receiving chemoconditioning compared with TBI (38 vs. 17%, respectively; *p* = not significant) (46). Notably, the relapse incidence for children who received RIC (*n* = 41) was similar to myeloablative chemotherapy (i.e., 38% for both groups) whilst NRM was lowest in the children who received RIC compared with those who received myeloablative chemotherapy or TBI (7.7 vs. 17.5 vs. 18.4%, respectively). These data, however, should be interpreted with caution as children who were selected to receive RIC may have clinical conditions precluding myeloablative chemo-conditioning and/or TBI, leading to bias. Comparative trials to assess RIC and myeloablative conditioning in patients eligible to receive either conditioning type are needed to determine the role of RIC regimens in hHSCT for childhood ALL.

## Strategies to Improve hHSCT Outcome

Progressive disease and infectious complications remain the leading causes of death after HSCT in paediatric ALL. Intervention strategies to reduce post-transplant relapse risk include improving conditioning regimens to exert more anti-neoplastic activity without additional toxicity, graft selection and engineering to augment GVL, and post-transplant chemotherapy to eliminate residual tumour cells (79). Newer strategies have focused on the modulation of donor-derived immune cells to harness the effect of GVL after transplant to prevent relapse without the side effects of GvHD. In some settings, adoptive immunotherapy has been used prophylactically, although this is more difficult to apply routinely as it is labour intensive and time consuming (80). In T-cell-depleted hHSCT, more advanced graft manipulation has been achieved with better understanding of pathophysiology in order to enhance the GVL effect.

Several mechanisms of leukaemia relapse have been described, and common to these is an acquired ability of malignant cells to escape immune surveillance through intrinsic or extrinsic driven processes. A well-described mechanism after HSCT involves tumour cells demonstrating copy neutral loss of heterozygosity of mismatched HLA haplotype on chromosome 6p by acquired somatic uniparental disomy, described as “genomic HLA loss” (81, 82). The resultant HLA alteration provides the tumour cells with the ability to evade patrolling donor T cells whose alloreactivity and overall GVL effect is mediated by the expression of mismatched HLA molecules on the surface of leukaemic cells. An important clinical implication for patients who develop HLA loss as a mechanism of relapse is the futility of administering additional donor T cells at relapse, given the lack of an HLA-mismatched target on tumour cells. Instead, a second allogeneic HSCT from a different donor would be useful to target the remaining HLA haplotype. Other relapse mechanisms that have been described include: (1) downregulation of HLA class II molecules, impairing the effects of donor T-cell alloreactivity that respond to HLA class II restricted peptides; (2) upregulation of inhibitory ligands by cancer cells, such as PD-L1 and B7-H3 (with the former associated with impairment of T-cell function); and (3) the release of immune-suppressive cytokines from tumour cells (IL-10, TGF-B) that upregulate the Treg population and inhibit T-cell and antigen-presenting cell function (81, 82).

The determinants for risk of relapse are multifactorial and dependent on various patient and treatment variables including biologic characteristics and disease risk before HSCT, conditioning intensity, and GvHD prophylaxis strategies. Additionally, issues to take into account include feasibility, tolerability and treatment toxicity, complications of opportunistic infections and GvHD (83). Targeted agents against an identified genomic mutation may be used as maintenance therapy to reduce residual tumour cells and prevent relapse after HSCT. Post-transplant use of a tyrosine kinase inhibitor against *BCR-ABL1* in patients with Ph^+^ ALL may reduce the risk of relapse, but this is not a consistent finding (81). In a prospective, multicentre study from 2013 involving 55 adult patients with Ph+ ALL randomised to receive imatinib pre-emptively or prophylactically, low rates of relapse were observed in both groups regardless of timing of therapy, and no significant differences in overall outcomes were observed between groups (84). In 2016, the Acute Leukaemia Working Party of the EBMT issued a recommendation to support either prophylactic or pre-emptive tyrosine kinase inhibitor therapy in patients with Ph^+^ ALL (85).

**Figure 1:**
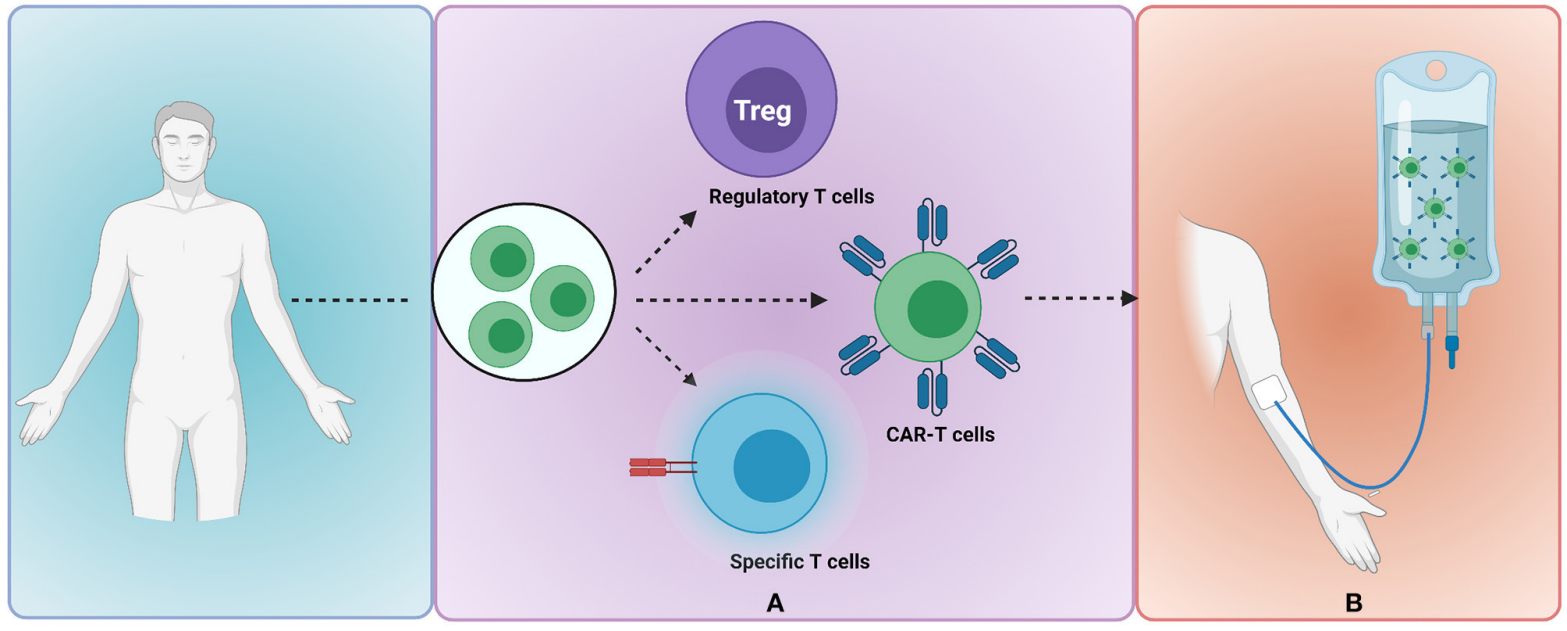
Donor-recipient immunocellular therapy to mitigate risk of leukaemia relapse. **(A)** Donor-derived T cells are selected and undergo modification or engineering to produce clonally expanded T cells of a specified subset e.g., regulatory T cells, CAR-T cells or T cells with externally inducible safety switch **(B)** adoptive transfer of modified T cells to the recipient. Image created with BioRender.com. CAR, chimeric antigen receptor.

Unique to hHSCT is the opportunity to exploit donor–recipient immunocellular mismatches to enhance GVL effects. Cellular therapy may be polyclonal and non-specific, as is the case of donor lymphocyte infusion (DLI), or may be engineered to target specific leukaemic cells (Figure 1).

In DLI, non-tolerant donor T cells are harnessed to augment the GVL effect and thus reduce the risk of overt relapse in states of mixed chimerism or positive MRD in patients with haematological malignancies. However, the effectiveness of DLI is not uniform across all haematological malignancies: better efficacy has been demonstrated for low-risk disease malignancies such as CML, chronic lymphocytic leukaemia, or low-grade lymphoma compared with high-risk malignancies such as ALL and AML (86). The use of DLI has been particularly disappointing in ALL as it does not consistently induce remission and is associated with risks of acute GvHD in 40–60% of patients, resulting in significant mortality (87–89). As earlier stated, DLI is ineffective in patients who demonstrate genomic HLA loss as a mechanism of relapse, and thus testing for HLA loss in patients who have relapsed is useful prior to employing DLI therapy.

CAR T-cell therapy is an established form of immunotherapy for relapsed/refractory B-cell precursor ALL; it is capable of inducing high remission rates but is associated with poor long-term LFS in the adult population (89). CAR T-cell therapy consolidated with later HSCT is associated with more durable remission compared with CAR T-cell therapy alone (90, 91). In a study involving 110 high-risk ALL patients of whom 42 had MRD-positive disease, CAR T-cell therapy cleared MRD in all and 73.5% of patients subsequently underwent allogeneic HSCT with a resultant 1-year EFS of 76.9% (92). Paediatric patients and young adults with high-risk ALL, however, display better long-term remission compared with adults and without the need for HSCT consolidation, with a 1-year EFS of 50%, calling into question which patients should be consolidated with HSCT after CAR T-cell therapy (93). In adults with high-risk ALL, Jiang et al. proposed several factors for consideration of consolidative HSCT after CAR T-cell therapy, including high-risk disease features pre CAR T-cell therapy, lymphodepletion without fludarabine, low persistence of CAR T-cells and B-cell recovery, and presence of a leukaemic sequence identified through next generation sequencing after CAR T-cell therapy (94). In the post-transplant setting, CAR T-cell therapy derived from either the donor or recipient may be used to treat relapse or used as prophylaxis against relapse. When applied to treat post-HSCT relapse, CAR T-cell therapy is able to induce high remission rates with a relatively low incidence of GvHD (<10%). Newer methods of applying CAR T cells as prophylaxis against relapse after HSCT in patients with ALL have been attempted. In China, two adult patients with high-risk ALL received infusion of donor-derived CD19^+^-CAR T cells 60 days after hHSCT as prevention against relapse (95). One patient had attained MRD-negative remission prior to HSCT and was disease free 1 year after HSCT. The other patient had undergone HSCT without achieving CR status; this patient attained MRD negativity after HSCT and remained disease free for 6 months. The long-term outcome of prophylactic CAR T-cell therapy after HSCT remains to be seen. Presently, CAR T-cell therapy may be used as a bridge to HSCT in selected paediatric patients with high-risk ALL in order to attain MRD-negative status and a better subsequent LFS. Alternatively, CAR T cells may be applied in the post-transplant setting to treat disease relapse or as prophylaxis against relapse in those deemed at highest risk (92).

Treg infusion in HSCT is associated with a reduced risk of GvHD without an increased risk of relapse and with improved immune reconstitution. Tregs counteract the effector T-cell alloreactivity that contributes to GvHD without inhibiting conventional T-cell cytotoxicity against cancer cells (18). The first study to describe adoptive transfer of Tregs in humans involved 28 patients with high-risk malignancies (5 with ALL) who underwent hHSCT. In that study, Di Ianni et al. showed that infusion of thymic-derived CD4^+^CD25^+^FoxP3^+^ Tregs on day−4 followed by CD34^+^-selected peripheral blood stem cells and conventional T-cell infusion on day 0 eliminated GvHD without the use of post-transplant immunosuppression; it also improved immune recovery and was not associated with an increased risk of relapse (96). A follow-up study by Martelli in 2014 further showed that use of adoptive immunotherapy with Tregs and conventional T cells was associated with a significantly reduced CIR (5 vs. >30%, respectively), and a trend towards better survival compared with historical controls (18). The transfer of Tregs together with a T-cell-replete graft containing conventional T cells results in the reduced incidence of GvHD and CIR and faster immune reconstitution with a broad T-cell repertoire (18, 80, 96). Tregs have not been associated with inhibition of general immunity or impaired responses to pathogens but rather promote stronger and faster immune reconstitution compared with historical controls (18, 80). The use of adoptive Treg transfer has also been associated with a broader T-cell repertoire upon reconstitution, increased frequency of pathogen-specific CD4/CD8 at 2 months (97), and improved immunity to opportunistic pathogens (96).

Newer cellular engineering modalities have also enabled the development of donor T cells with improved specificity to accelerate engraftment and immune reconstitution, target leukaemic cells to reduce relapse risk, and improve infective immunity. To abrogate the risk of uncontrolled GvHD brought by donor T cell add-back, these cells may be transduced with a safety switch that is externally inducible in the event of GvHD. The first study to assess the efficacy of the inducible caspase 9 (*iCasp9*) suicide gene in hHSCT was by Di Stasi et al. In this study, five children who had undergone hHSCT for relapsed acute leukaemia received an infusion of donor T cells expressing *iCasp9*. Following *iCasp9* induction, more than 90% of the modified T cells were eliminated and there was rapid resolution of GvHD (98). Another study reported on the long-term outcome of HSCT with *iCasp9*-transduced T cells in 10 patients with haematological malignancies; these patients demonstrated long-term persistence of the modified T cells *in vivo*, with immune benefit that was conferred in both the early phase, by the infused cells themselves, and in the later phase, through rapid reconstitution of naïve T lymphocytes, thus providing sustained immune protection against viral pathogens (99).

In the setting of hHSCT, donor–recipient alloreactive NK-cell mismatch can mediate killing of residual tumour cells through the presence of inhibitory receptors on single KIR donor NK cells that bind ligands present in the donor and absent in the recipient; this is known as the “missing self” theory (100, 101). As described earlier, NK-cell alloreactivity enhances anti-leukaemic effect without mediating GvHD. The clinical utility of NK-cell alloreactivity is dependent on the transplant platform used; more beneficial effects have been documented in the context of T-cell-depleted hHSCT for acute leukaemia rather than T-cell-replete hHSCT with PTCy, although data showing benefit have been more consistent for AML than ALL. Outcomes of NK-cell alloreactivity have a lesser impact in non-myeloablative-based hHSCT-PTCy due to the over-riding effects of T-cell immunosuppressive therapy. PTCy selectively and completely eliminates actively proliferating NK cells derived from the graft, impairs NK-cell recovery and maturation, and negates the overall impact of NK-cell KIR ligand mismatches on HSCT outcome (102).

An area of interest for future research is the use of specific cytokines to promote polyclonal expansion of haematopoietic stem cells to improve immune reconstitution, reduce rates of infection and reduce the risk of relapse. Infusion of mature donor alloreactive NK cells with the addition of IL-15 for *in vivo* expansion of NK cells reduces the incidence of relapse and viral infections (102). IL-15 promotes the expansion of T and B cells and the survival of NK cells as well as promoting the generation of CD8^+^ memory T cells (82). IL-2 given at low doses can promote the proliferation of T, B and NK cells and restore haemostasis of CD4^+^ T cells and Tregs, improving T-cell reconstitution and GVL effect without increased GvHD risk (97). Interferon alpha has direct anti-tumour activity, enhances NK-cell cytotoxicity and stimulates dendritic cells important in immune surveillance and the directed killing of malignant cells (82).

In summary, strategies incorporating cell- and immune-based immunotherapy after HSCT provide the opportunity to enhance GVL effect, reduce the risk of relapse, improve immune reconstitution, reduce rates of infection and reduce the risk of severe GvHD. Post-transplant maintenance chemotherapy, such as tyrosine kinase inhibitors in patients with Ph^+^ ALL, has also been shown to be useful.

## Conclusion

hHSCT represents a promising therapeutic approach for children with ALL who require HSCT but lack an HLA-matched donor. The exponential increase in the use of hHSCT for haematological malignancies in the last 10 years has allowed more data to emerge from the paediatric ALL population to guide optimal management choices. Studies to date have shown comparable OS and EFS in children who have undergone hHSCT for ALL in CR1/CR2 with those who underwent HSCT from an MSD or MUD, although survival rates remain poor for those transplanted in advanced or active disease. Preparatory regimens containing TBI are currently recommended for children and adolescents with ALL based on the results of several large studies reporting superior EFS and CIR with TBI-based conditioning compared with chemo-conditioning alone. The criteria for selection of a haploidentical family donor according to the different transplant platforms used has been further refined with better understanding of the donor–recipient immune interactions that underpin the GVL effect and mediate GvHD. Strategies to reduce relapse risk after hHSCT have focused on newer cellular-based therapies to harness the GVL effect without increasing the incidence of GvHD and overall NRM. Lastly, the ability to perform HSCT with reasonably good outcomes, unrestricted by the HLA barrier, has significantly expanded donor choices and may address ethical issues related to using minor siblings as donors for children with ALL.

## Author Contributions

SA, TM, MY, and HA contributed to the concept and design of this review, to the assembly, analysis and interpretation of data, and to manuscript writing and final approval. All authors are accountable for all aspects of this work.

## Funding

HA and SA are supported by University of Malaya IIRG Grant (No.021-2019).

## Conflict of Interest

The authors declare that the research was conducted in the absence of any commercial or financial relationships that could be construed as a potential conflict of interest. The handling editor declared a shared consortium with several of the authors HA and TM at time of review.

## Publisher's Note

All claims expressed in this article are solely those of the authors and do not necessarily represent those of their affiliated organizations, or those of the publisher, the editors and the reviewers. Any product that may be evaluated in this article, or claim that may be made by its manufacturer, is not guaranteed or endorsed by the publisher.
